# Cost-effectiveness analysis of treatment with peginterferon-alfa-2a versus peginterferon-alfa-2b for patients with chronic hepatitis C under the public payer perspective in Brazil

**DOI:** 10.1186/1478-7547-11-25

**Published:** 2013-10-08

**Authors:** Fabio MR Barros, Hugo Cheinquer, Carolina T Tsuchiya, Eduardo AV Santos

**Affiliations:** 1 Hospital Português de Beneficência em Pernambuco e Hospital das Clínicas – UFPE, Recife, PE, Brazil; 2 Hospital das Clínicas da Universidade Federal do Rio Grande do Sul, Porto Alegre, RS, Brazil; 3 Roche Brazil, São Paulo, SP, Brazil

**Keywords:** Chronic hepatitis C, Peginterferon-alfa-2a, Peginterferon-alfa-2b, Cost-effectiveness, Brazil, SUS

## Abstract

**Background:**

Chronic hepatitis C affects approximately 170 million people worldwide, and thus being one of the main causes of chronic liver disease. About 20% of patients with chronic hepatitis C will develop cirrhosis over 20 years, and present an increased risk of developing hepatic complications. Sustained virological response (SVR) is associated with a better prognosis compared to untreated patients and treatment failures.

The objective of this analysis was to compare treatment costs and outcomes of pegylated interferon-alfa-2a *versus* pegylated interferon-alfa-2b, both associated with ribavirin, in the therapeutic scheme of 24 weeks and 48 week for hepatitis C genotypes 2/3 and genotype 1, respectively, under the Brazilian Public Health System (SUS) scenario.

**Methods:**

To project disease progression, a Markov model was built based on clinical stages of chronic disease. A Delphi panel was conducted to evaluate medical resources related to each stage, followed by costing of related materials, services, procedures and pharmaceutical products. The evaluation was made from a public payer perspective. The source used for costing was government reimbursement procedures list (SAI/SIH–SUS). Drug acquisition costs were obtained from the Brazilian Official Gazette and “Banco de Preços em Saúde” (government official source). It was assumed a mean patient weight of 70 kg. Costs were reported in 2011 Brazilian Reais (US$1 ≈ $Brz1.80). A systematic review followed by a meta-analysis of the 7 identified randomized controlled trials (RCTs) which compared pegylated interferons, was conducted for obtaining relative efficacy of both drugs: for genotype 2/3, mean rate of SVR was 79.2% for peginterferon-alfa-2a and 73.8% for peginterferon-alfa-2b. For genotype 1, SVR mean rate was 42.09% *versus* 33.44% (peginterferon-alfa-2a and peginterferon-alfa-2b respectively). Time horizon considered was lifetime. Discount rate for costs and outcomes was 5%, according to Brazilian guidelines for Health Technology Assessment (HTA).

**Results:**

Analysis showed that peginterferon-alfa-2a is a dominant therapy compared to peginterferon-alfa-2b for genotype 1 ($Brz 4,345 savings and 0.10 LY/0.25 QALY gains) as well for genotype 2/3 ($Brz 8,001 savings and 0.16 LY/0.39 QALY gains). Projections indicated that for each 1000 patients treated with peginterferon-alfa-2a instead of peginterferon-alfa-2b, the amount of resources saved would be of $Brz 4.3 million for genotypes 2/3 and up to $Brz 8 million for genotype 1.

**Conclusion:**

These findings suggest that treatment with peginterferon-alfa-2a is more effective and less costly when compared to peginterferon-alfa-2b under SUS perspective in Brazil.

## Background

Hepatitis C virus (HCV) identification is relatively recent (1989) [1] and many efforts were made in the last years to optimize its pharmacological treatment and disease detection rates.

HCV infection becomes chronic in approximately 75%–85% of cases, significantly raising healthcare costs, especially those resulting from hospital bills. According to Wong et al., infection with hepatitis C virus is the leading cause in approximately 30% of liver transplantation, 40% of cases of decompensated cirrhosis and 60% of hepatocellular carcinoma [2]. Sustained virological response (SVR) in chronic hepatitis C is associated with better prognosis, since observed reduction in clinical events, mainly liver failure is observed when SVR is achieved [3].

In 2010, Brazilian Ministry of Health published information on anti-HCV (antibody to the hepatitis C virus, its presence indicates an active or chronic hepatitis C infection) prevalence in Brazilian capital cities; estimated anti-HCV prevalence was 2.1% in North Region, 0.7% in Northeast Region, 1.3% in Central-West Region, 1.3% in Southeast Region and 1.2% in South Region [4]. A total of 60,908 cases of hepatitis C were confirmed and registered in Brazil from 1999 to 2009 according to 2010 Viral Hepatitis Epidemiological Bulletin [5].

According to Brazilian Ministry of Health, in 2011, expenses with Hepatitis C achieved around $Brz 17.7 million [6]. Moreover, disease’s chronic nature and its successive stages, such as cirrhosis, hepatocellular carcinoma or liver transplantation and negative issues produced by disease, such as absenteeism, early retirement, loss of productivity, aggravate social and economic burden related to HCV infection. In this scenario, the adoption of cost-effective strategies would beneficiate health policy makers, society and patients.

In the Public Healthcare System of Brazil (SUS), according to the therapeutic guidelines and clinical protocols for hepatitis C [7], patients with genotype 1 aged from 18 to 70 years old, with platelet counts > 90,000/mm^3^ (non cirrhotic) or > 75,000/mm^3^ (cirrhotic) after qualitative PCR (Polymerase Chain Reaction), should be treated with pegylated interferon alfa-2a or 2b weekly and ribavirin 15 mg/kg daily for 48-72-week treatment period. Patients with genotypes 2 or 3, in the absence of low SVR predictors – METAVIR score ≥ F3 or clinical manifestations of cirrhotic fibrosis or viral load > 600,000 UI/mL –, should be treated with conventional interferon alfa-2a or 2b, 3 times a week and ribavirin 15 mg/kg daily for 24-week treatment period; in the presence of low SVR predictors, patients should be treated with pegylated interferon alfa-2a or 2b weekly and ribavirin 15 mg/kg daily for 24-48-week treatment period. The guideline doesn’t mention any differences between efficacies of both pegylated interferons. However, literature has been discussing the clinical superiority of one of them, with the tendency to peginterferon-alfa-2a [8-13]. Economic assessment comparing peginterferon-alfa-2a and peginterferon-alfa-2b in Chronic Hepatitis C indicates that the first one was considered cost-effective or even dominant in several countries like: USA, UK, Spain, Poland and Mexico [14-20].

The objective of this analysis was to assess the cost-effectiveness ratio of peginterferon-alfa-2a *versus* peginterferon-alfa-2b both plus ribavirin (RBV), in a 24-week therapeutic schedule in the treatment of patients with chronic hepatitis C, genotypes 2 and 3 or 48-week schedule for genotype 1, from the perspective of the Public Health Care System in Brazil. In order to obtain efficacy data for the analysis, our aim was also to conduct a systematic review of RCTs and perform a meta-analysis of the results.

## Methods

We used a Markov model to simulate the progression of chronic hepatitis C in a hypothetical cohort of patients with initial age of 45 years old presenting antibodies against hepatitis C virus (anti-HCV) or HCV RNA positivity in individuals known to be previously negative. The model predicted long-term clinical benefits (Life Years - LYs and Quality Adjusted Life Years - QALYs) and associated costs with each intervention, providing the incremental cost-effectiveness ratio (ICER) of peginterferon-alfa-2a *versus* peginterferon-alfa-2b, both associated with ribavirin. A one-way sensitivity analysis was also conducted in order to assess the impact of uncertainty on the cost-effectiveness ratio.

### Model description

A Markov model was used as it allows representing the relevant stages of the natural history of the disease over time, as well as estimates for probability of progression between the several health states related to the chronic infection with hepatitis C virus.

Each scenario was comprised by 7 mutually excluding clinical stages, each of which was defined by a patient’s clinical condition: sustained virological response, chronic hepatitis C, compensated cirrhosis, decompensated cirrhosis, hepatocellular carcinoma, liver transplantation and death, enabling the comparison of final outcomes between therapeutic alternatives.

Associated with each of the clinical stages, quality-of-life values and costs were attributed. Costs associated to Markov stages intended to reflect local medical practice, and resource utilization was determined by a Delphi panel. The panel included prescribing professionals from the public healthcare system. All costs and outcomes were discounted at a 5% rate, according to the Brazilian Guidelines for Health Technology Assessment (HTA) [21].

### Diagram of transition between stages

**Figure 1:**
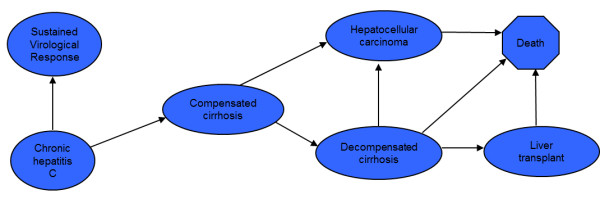
Diagram for patients chronically infected with hepatitis C virus: Diagrams of transition for treatment of chronic infection with hepatitis C virus in mutually excluding stages.

Following, we present diagrams of transition for treatment of chronic infection with hepatitis C virus in mutually excluding stages (Figure 1). Consequently, each patient will correspond to one of the stages at a given time.

The model was similar to others described in literature [22-24]. Patients start at the chronic hepatitis C stage, undergo a 24-weeks treatment with peginterferon-alfa-2a plus ribavirin or peginterferon-alfa-2b plus ribavirin if genotypes 2/3 and 48-weeks treatment with the same drugs if genotype 1. No extension on treatment schedule was considered.

The time horizon of the analysis accounted for patient’s lifetime and was divided into equal increments of time, known as Markov cycles. Each cycle comprises one year and defines possible pathway for each patient in the model based on the natural history of the infection with hepatitis C virus. The probability for a patient to migrate between two stages during a cycle is called *probability of transition between stages*. Following 24 or 48 weeks of treatment, patients may show positive response to treatment or recurrence every year.

After the treatment cycle, patients can either respond to treatment, which means moving to sustained virological response stage, remain at the chronic stage or progress to compensated cirrhosis. When at the compensated cirrhosis, patients can remain on the stage or progress either to hepatocellular carcinoma or decompensated cirrhosis. Patients who develop decompensated cirrhosis could remain on the stage, develop hepatocellular carcinoma, receive a liver transplantation or have an increased risk disease-related mortality. Finally, patients at the liver transplantation stage or hepatocellular carcinoma can remain on the stage or die.

### Transition probabilities

We reviewed clinical and pharmacoeconomic literature aiming to identify studies reporting probability of clinical events which were relevant for the model. These data are presented in the Table 1[25-34].

**Table 1:** Transition probabilities employed in the model

**Health status**		**Transition Probability (annual)**
**From**	**To**	**Rate**
Chronic hepatitis C	Compensated cirrhosis [25,26]	0.073
Compensated cirrhosis	Decompensated cirrhosis [27,28]	0.039
	Hepatocellular carcinoma [25,29,30]	0.014
Decompensated cirrhosis	Hepatocellular carcinoma [25,27,28]	0.014
	Liver transplant (1st year) [31]	0.031
	Death [24,25,29]	0.129
Hepatocellular carcinoma	Death [25]	0.427
Liver transplant	Death (1st year) [24,29,32-34]	0.210
Liver transplant	Death (> 1 year) [24,29-32]	0.057

The transition probabilities were employed as model parameters for each of the stages of chronic hepatitis C, compensated cirrhosis, decompensated cirrhosis, hepatocellular carcinoma, liver transplantation, post-transplantation and death. All patients had their risk of death adjusted by the Brazilian Agency for Geography and Statistics (IBGE) life table, which provides mortality rates for the Brazilian population [35].

### Quality of life

Relevant data concerning quality-of-life values (utilities) were extracted from the international literature, due to the lack of local data [24,29,36]. The utilities weights assigned for each stage were: 0.90 for sustained virological response, 0.82 for chronic hepatitis C, 0.78 for compensated cirrhosis, 0.65 for decompensated cirrhosis, 0.25 for hepatocellular carcinoma, 0.5 for liver transplant at the 1^st^ year, 0.7 for the subsequent years after liver transplant and finally 0 for death.

### Cost data

This study was performed using a public payer’s. Annual medical resources related to each clinical stage of chronic hepatitis C were collected through a Delphi panel, which included experts in infectious diseases and hepatology with 10 to 24-year experience in medical practice. Source used for costing was government reimbursement procedures list (SAI/SIH–SUS) [37]. The aggregated annual costs of managing each clinical stage are shown in Table 2.

**Table 2:** Medical costs for treating several stages of chronic infection with hepatitis C virus

**Cost item**	**Cost data**
	**Cost / year / patient**
Chronic hepatitis C	$Brz 586
Compensated cirrhosis	$Brz 852
Decompensated cirrhosis	$Brz 21,716
Hepatocellular carcinoma	$Brz 12,526
Liver transplant, 1st year	$Brz 63,013
Liver transplant, subsequent year	$Brz 7,777

Notes: Costs based on SAI/SIH–SUS for procedures and Brazilian Official Gazette or “Banco de Preços em Saúde” for drugs.

Since in Brazil peginterferon-alfa drugs are acquired in a centralized purchasing process, their acquisition costs were obtained from purchase invoices published in the Brazilian Official Gazette [38-40]. Ribavirin acquisition cost was taken from “Banco de Preços em Saúde” (BPS), the government official source. Peginterferon-alfa 2a is given at a dosage of 180 μg per week irrespectively of patient weight and has only one presentation. However, for peginterferon-alfa 2b, dosage is based on patient’s weight (1.5 μg/kg/week) and has three distinct forms of presentation with different prices per μg. As specified in the peginterferon-alfa 2b SPC (summary of product characteristics), we have considered that unused portion of drug was wasted. Therefore, it was assumed a mean patient weight of 70 kg (which means 105 μg of peginterferon-alfa 2b) and the weighted average per μg available at BPS was used. For ribavirin, recommended dosage is 15 mg/kg/day. Costs were reported in 2011 Brazilian Reais (US$1 ≈ $Brz1.80) (Table 3).

**Table 3:** Weekly cost for both pegylated interferons

	**Peginterferon-alfa-2a**	**Peginterferon-alfa-2b**
Drugs cost	180 μg /week – $Brz 269.09	105 μg /week –$Brz 381.47
Ribavirin cost per week	$Brz 16.72	$Brz 16.72
Weekly cost	$Brz 285.82	$Brz 398.20

### Efficacy data

Efficacy data for pegylated interferons, both combined with ribavirin, used in this cost-effectiveness study were derived from a meta-analysis conducted in order to assess relative treatment efficacy of peginterferon-alfa-2a compared to peginterferon-alfa-2b both plus ribavirin. Before carrying out the meta-analysis, a systematic review of literature was performed.

An extensive search through main medical information databases (MEDLINE, Cochrane Library, Embase and Lilacs) and specialized websites was conducted on the second trimester of 2010. We aim to identify RCTs which evaluated treatment efficacy of peginterferon-alfa-2a *versus* peginterferon-alfa-2b both plus ribavirin for HCV treatment in patients not co-infected with HIV (Table 4).

**Table 4:** Specific search strategy in databases

**Search**	**Database**	**Search Terms**	**Results**
1	Medline	Exp hepatitis C.mp. and exp peginterferon alfa 2a.mp. and exp peginterferon alfa 2b.mp. NOT HIV[Title]	475
2	Pubmed	Exp hepatitis C.mp. and exp peginterferon alfa 2a.mp. and exp peginterferon alfa 2b.mp. NOT HIV[Title]	402
3	Embase	Exp hepatitis C.mp. and exp peginterferon alfa 2a.mp. and exp peginterferon alfa 2b.mp.	222
4	Cochrane	Exp hepatitis C.mp. and exp peginterferon alfa 2a.mp. and exp peginterferon alfa 2b.mp. NOT HIV[Title]	42
5	LILACS	Exp hepatitis C.mp. and exp peginterferon.mp.	12
6	Hand search	Internet searches and targeted searches in specialist liver websites and associations	4

Notes: exp, explode—a search term that automatically includes closely related indexing terms; mp, multiple posting—term appears in title, abstract, or subject heading.

After removing duplicates, all studies tittles were screened and potentially relevant references were selected by two independent reviewers. Abstract and full text of the potential articles were also analyzed and the selection of studies was made. Included studies were those which evaluate treatment efficacy of peginterferon alfa-2a versus peginterferon alfa-2b both plus ribavirin for hepatitis C treatment in naïve patients or nonresponders to other therapies not co-infected with HIV (Table 5).

**Table 5:** Study inclusion criteria

**Criteria**	**Definition**
Population	Patients infected with hepatitis C virus (HCV) not co-infected with HIV.
Intervention	Pegylated interferon alfa – 2a plus ribavirin (RBV)
Comparator	Pegylated interferon alfa – 2b plus ribavirin
Outcome	Sustained Virological Response (SVR)
Study type	Randomized controlled trials

To summarize all information gathered in our systematic review a meta-analysis was performed and according to heterogeneity test, a fixed or a random-effect model was considered.

### Sensitivity analysis

In order to assess the uncertainty of the results, a one-way sensitivity analysis was performed. The parameters peginterferon-alfa-2a cost, peginterferon-alfa-2b cost, state-transition probabilities; medical costs and utility values were varied over the range of 15% (up and down); patient weight was varied from 50 to 90 kg; SVR was varied according to lower and upper limit values obtained from meta-analysis and starting age was varied from 40 to 50 years old. Sensitivity analysis within same conditions was conducted for cost-effectiveness analysis of genotype 1 and genotype 2/3.

## Results

### Meta-analysis

**Figure 2:**
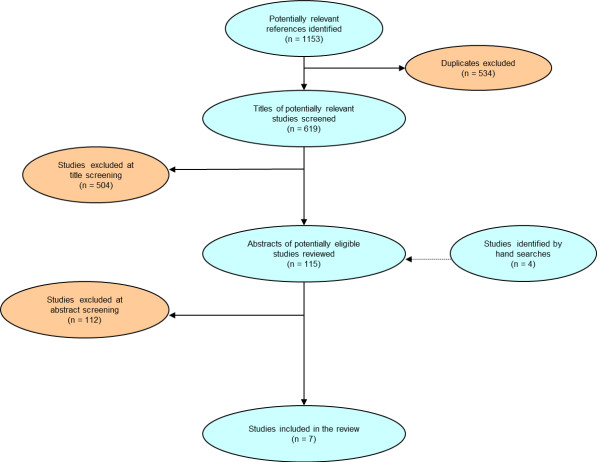
Study selection flow chart: Seven papers out of 623 found (databases citation without duplicates plus manual searches) which met our inclusion criteria were included in meta-analysis.

We identified seven papers out of 623 found (databases citation without duplicates plus manual searches) which met our inclusion criteria described above: 1) Ascione, 2009 [41] 2) Rumi, 2009 [42] 3) McHutchison, 2009 [43] 4) Yenice, 2006b [44] 5) Scotto, 2008 [45] 6) Berak, 2007 [46] and 7) Kolakowska, 2008 [47]. Not all papers evaluated all patients’ genotypes, therefore, for genotype 2/3, four papers were included in the meta-analysis and for genotype 1, six papers were evaluated. All studies were prospective randomized controlled trials (Figure 2).

**Figure 3:**
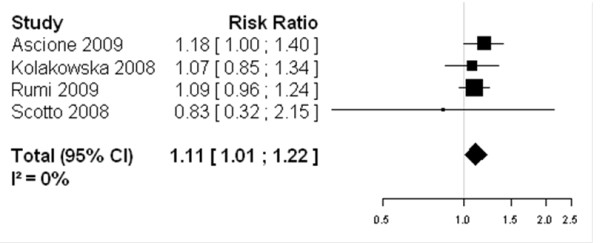
Forest Plot of studies comparing the effect of treatment with peginterferon-alfa-2a vs standard-dose peginterferon-alfa-2b for genotypes 2/3: Peginterferon-alfa-2a showed higher SVR as compared to peginterferon-alfa-2b: 79.2% vs 73.8% (RR = 1.11, IC 95% 1.01 – 1.22, assuming a fixed-effect model).

**Figure 4:**
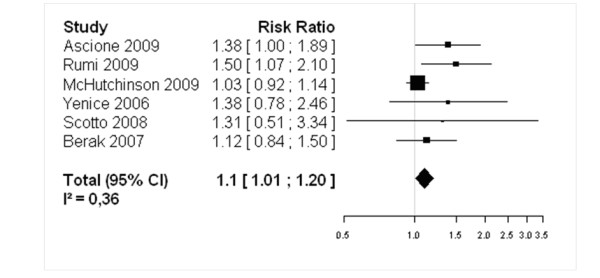
**Forest Plot of studies comparing the effect of treatment with peginterferon-alfa-2a vs standard-dose peginterferon-alfa-2b for genotype 1: Peginterferon-alfa-2a showed higher SVR as compared to standard dose of peginterferon-alfa-2b as well: 42.09% *versus *33.44% (RR = 1.10, IC 95% 1.01 – 1.20, assuming a fixed-effect framework).**

For genotypes 2/3 peginterferon-alfa-2a showed higher SVR as compared to peginterferon-alfa-2b: 79.2% versus 73.8% (RR = 1.11, IC 95% 1.01 – 1.22, assuming a fixed-effect model) (Figure 3); for genotype 1 chronic HCV infection patients, peginterferon-alfa-2a showed higher SVR as compared to standard dose of peginterferon-alfa-2b as well: 42.09% *versus* 33.44% (RR = 1.10, IC 95% 1.01 – 1.20, assuming a fixed-effect framework) (Figure 4). These findings suggest that peginterferon-alfa-2a is associated with a higher clinical response than peginterferon-alfa-2b.

It is important to mention that only standard-dose peginterferon-alfa-2b data were included in the meta-analysis. Therefore, the effect of the low-dose: 1.0 μg/kg of peginterferon-alfa-2b, analyzed in McHutchison [43], 2009 was excluded from the analysis in order to avoid a possible negative impact over the results.

On the other hand, including peginterferon-alfa-2b lower dose appear not to influence the results. A recently published meta-analysis [8] (Awad, 2010) that did not exclude the effects of low-dose treatment showed similar results of efficacy of peginterferon-alfa-2a vs peginterferon-alfa-2b presented in this study. For genotype 1, peginterferon-alfa-2a showed higher sustained virological response when compared to peginterferon-alfa-2b: 42.1% versus 33.3% (RR = 1.11, IC 95% 1.02 – 1.20, assuming a fixed-effect model).

### Cost-effectiveness analysis

Our model predicted the costs and outcomes of each alternative by combining the treatment expenses with the costs related to each future stage of the disease progression.

### Genotypes 2/3

Overall cost for treatment with peginterferon-alfa-2a was $Brz 13,120, while for peginterferon-alfa-2b, total cost was $Brz 17,465. Treatment with peginterferon-alfa-2a resulted in 15.21 LYs and 14.57 QALYs, while treatment with peginterferon-alfa-2b resulted in 15.11 LYs and 14.32 QALYs. Clinical and economic results are presented in Table 6 for 24 weeks of treatment with peginterferon-alfa-2a or peginterferon-alfa-2b, both in association with a ribavirin.

**Table 6:** Comparative chart of clinical and economic results for treatment of patients with hepatitis C genotype 2/3

**Therapeutic schedule**	**Total costs**	**LY**^**1**^	**QALY**^**2**^	**Result**
Peginterferon-alfa-2a + ribavirin	$Brz 13,120	15.21	14.57	peginterferon-alfa-2a is dominant
Peginterferon-alfa-2b + ribavirin	$Brz 17,465	15.11	14.32	

Notes: ^1^LYs – Life years; ^2^QALYs – Quality Adjusted Life Years.

In this setting, peginterferon-alfa-2a is a dominant therapy compared to peginterferon-alfa-2b, i.e. less costly ($Brz 4,345 savings) and more effective (0.10 LY and 0.25 QALY gains).

### Genotype 1

Overall cost for the treatment with peginterferon alfa-2a was $Brz 31,185, while for peginterferon alfa-2b, total cost was $Brz 39,186. Peginterferon-alfa-2a therapy resulted in 14.51 LY and Clinical and economic result is presented in Table 7 for 48 weeks of treatment with peginterferon alfa-2a or peginterferon alfa-2b, both in association with a ribavirin.

**Table 7:** Comparative chart of clinical and economic results for treatment of patients with hepatitis C genotype 1

**Therapeutic schedule**	**Total costs**	**LY**^**1**^	**QALY**^**2**^	**Result**
Peginterferon-alfa-2a + ribavirin	$Brz 31,185	14.51	12.89	peginterferon-alfa-2a is dominant
Peginterferon-alfa-2b + ribavirin	$Brz 39,186	14.35	12.50	

Notes: ^1^LYs – Life years; ^2^QALYs – Quality Adjusted Life Years.

Peginterferon-alfa-2a is a dominant therapy also for this genotype, with lower related costs ($Brz 8,001 savings) and higher effectiveness (0.16 LY and 0.39 QALY gains).

### Sensitivity analysis

By observing the results from the one-way sensitivity analysis, in Tables 8 and 9, peginterferon-alfa-2a was a dominant therapy in all scenarios: the drug yielded gains in LYs and QALYs although incurred lower costs as opposed to peginterferon-alfa-2b. For genotype 2/3, variables with highest impact were patient weight, sustained virological response and utility values. For genotype 1, variables with highest impact were sustained patient weight, utility values and peginterferon-alfa-2b acquisition cost.

**Table 8:** One-way sensitivity analysis results for genotype 2/3 comparing peginterferon-alfa-2a to peginterferon-alfa-2b

**Parameter**	**Lower parameter value**			**Higher parameter value**			**Result**
	**Cost difference**	**LY difference**	**QALY difference**	**Cost difference**	**LY difference**	**QALY difference**	
Utility values^1^	-$Brz 4345	0,10	0,34	-$Brz 4345	0,10	0,17	Dominant
Medical costs^1^	-$Brz 4098	0,10	0,26	-$Brz 4592	0,10	0,26	Dominant
State-transition probabilities^1^	-$Brz 4250	0,08	0,22	-$Brz 4411	0,13	0,27	Dominant
Peginterferon-alfa-2a acquisition cost^1^	-$Brz 5314	0,10	0,26	-$Brz 3376	0,10	0,26	Dominant
Peginterferon-alfa-2b acquisition cost^1^	-$Brz 2972	0,10	0,26	-$Brz 5718	0,10	0,26	Dominant
Patient weight^2^	-$Brz 1729	0,10	0,26	-$Brz 6961	0,10	0,26	Dominant
SVR^3^	-$Brz 3576	0,05	0,14	-$Brz 5114	0,15	0,38	Dominant
Starting age^4^	-$Brz 4459	0,13	0,29	-$Brz 4927	0,09	0,22	Dominant

Notes: ^1^Parameters were varied over the range of ±15%; ^2^Parameter was varied from 50 to 90 kg; ^3^Parameter was varied according to lower and upper limit values obtained from meta-analysis; ^4^Parameter was varied from 40 to 50 years old.

**Table 9:** One-way sensitivity analysis results for genotype 1 comparing peginterferon-alfa-2a to peginterferon-alfa-2b

**Parameter**	**Lower parameter value**			**Higher parameter value**			**Result**
	**Cost difference**	**LY difference**	**QALY difference**	**Cost difference**	**LY difference**	**QALY difference**	
Utility values^1^	-$Brz 8001	0,16	0,54	-$Brz 8001	0,16	0,27	Dominant
Medical costs^1^	-$Brz 7610	0,16	0,41	-$Brz 8392	0,16	0,41	Dominant
State-transition probabilities^1^	-$Brz 7851	0,12	0,37	-$Brz 8105	0,20	0,44	Dominant
Peginterferon-alfa-2a acquisition cost^1^	-$Brz 9938	0,16	0,41	-$Brz 6063	0,16	0,41	Dominant
Peginterferon-alfa-2b acquisition cost^1^	-$Brz 5254	0,16	0,41	-$Brz 10747	0,16	0,41	Dominant
Patient weight^2^	-$Brz 2769	0,16	0,41	-$Brz 13232	0,16	0,41	Dominant
SVR^3^	-$Brz 7902	0,16	0,39	-$Brz 8099	0,17	0,42	Dominant
Starting age^4^	-$Brz 8181	0,20	0,45	-$Brz 7788	0,14	0,37	Dominant

Notes: ^1^Parameters were varied over the range of ±15%; ^2^Parameter was varied from 50 to 90 kg; ^3^Parameter was varied according to lower and upper limit values obtained from meta-analysis; ^4^Parameter was varied from 40 to 50 years old.

Even considering a hypothetical scenario for genotype 2/3 with 50 kg patients, where peginterferon-alfa-2a cost was simultaneously increased by 15% and peginterferon-alfa-2b cost was simultaneously decreased by 15%, peginterferon-alfa-2a would still be very cost-effective, resulting in a low incremental cost-effectiveness ratio of $Brz 894 per QALY.

## Discussion

The results obtained from the meta-analysis are in line with other six recently published meta-analyses (Awad, 2010 [8]; Alavian, 2010 [9]; Zhao, 2010 [10]; Xiao, 2010 [11]; Singal, 2011 [12] and Cheinquer, 2010 [13]) which concluded that peginterferon-alfa-2a has a higher SVR as compared to peginterferon-alfa-2b, with similar safety profile.

This work applied a Markov model to represent the treatment course of the disease, as well as to estimate the cost-effectiveness ratio of the therapeutic schedule with peginterferon-alfa-2a associated with ribavirin, when compared to peginterferon-alfa-2b also associated with ribavirin, aiming to evaluate which drug would represent more advantages to the Brazilian public healthcare system.

The model evaluated 2 different treatment regimens according to patients genotype: patients diagnosed with genotypes 2 and 3 would be eligible to receive 24 weeks of treatment, while patients with genotype 1 would receive 48 weeks of treatment.

It is important to note that analysis presents limitations regarding the usage of data from other countries to adapt transition probabilities and evaluate quality of life. The lack of Brazilian data should be supplied with more studies on this field, which would enable more reliable and accurate local economic analysis.

The extrapolation of results obtained in the analysis revealed that therapy using peginterferon-alfa-2a presented favorable results relative to health life years – LYs and QALYs, being dominant compared to peginterferon-alfa-2b, for a 24-week or 48-week treatment period.

Based on our results, the projections indicate that for each 1000 patients treated with peginterferon-alfa-2a instead of peginterferon-alfa-2b, the amount of resources saved by the SUS can be of up to $Brz 4.3 million for genotypes 2/3 and up to $Brz 8 million for genotype 1, and that would allow the treatment of approximately 145 and 108 more patients, respectively.

Finally, it is important to stress that this study, which enhances the available information on health economics field in Brazil, intends to support medical and health-care professionals, by providing adequate information in decision-making on hepatitis C, a chronic disease which requires special attention to be provided to patients and to the management of resources used in its treatment. Specially, with the introduction of new protease inhibitor drugs, telaprevir and boceprevir, the triple therapy (association of telaprevir or boceprevir with pegylated interferon and ribavirin) has been emerging as standard of care for chronic hepatitis C. The introduction of those new drugs significantly changes the landscape and costs of HCV management. In this new scenario, multiple factors should be considered. Adoption of cost-effective strategies would help decision makers to ensure appropriate usage of resources and proper treatment of patients.

## Conclusions

Our findings suggest that treatment with peginterferon-alfa-2a is associated with higher clinical responses and outcomes than peginterferon-alfa-2b. The pharmacoeconomic analysis assessed peginterferon-alfa-2a as a dominant therapy (more effective and less costly) as compared to peginterferon-alfa-2b under SUS perspective in Brazil.

## Competing interests

We declare that the article was funded by Hoffmann-La Roche Ltd. All authors have received financial sponsorship by Roche Pharmaceuticals. FMRB has also received financial support by Merck Sharp & Dohme, Bristol-Myers Squibb, Bayer HealthCare Pharmaceuticals and Biolab Sanus. HC has also received financial support by Bristol-Myers Squibb, Novartis and Janssen-Cilag.

## Authors’ contributions

Fabio Barros and Hugo Cheinquer provided all medical and scientific support. Carolina Tsuchiya was responsible for writing, analysis and revision. Eduardo Santos provided health economic expertise, designed the model structure and economic evaluations, and supervised the writing, analysis and revision. All authors read and approved the final manuscript.
